# Notes from the Congresses

**Published:** 1897-09-11

**Authors:** 


### Medical Progress and Hospital Clinics.

[*The Editor will be glad to receive offers of co-operation and contributions from members of the profession. All letters should be addressed to* The Editor, at the Office, 28 & 29, Southampton Street, Strand, London, W.C.]

### NOTES FROM THE CONGRESSES.

The two great Congresses which have recently been in session—the International Medical Congress in Moscow, and the British Medical Association in Montreal—have been well attended; and, although in the case of the former the heat was such as to quickly dissipate the audiences in many of the sections, the communications which were read or presented for publication have been in many cases of very great interest. The Congress met on August 19th, and its proceedings terminated on August 25th.

Hardly had the Congress of the East been brought to a close when the Congress of the West, that of the British Medical Association, opened its doors. The proceedings really commenced on Monday, August 30th, when Professor Roddick entertained a hundred guests, including the officers of the Association, at dinner to meet the Governor-General and other prominent Canadians. The same evening the Canadian Medical Association entertained the members generally at a smoking concert.

The meeting was formally inaugurated on August 31st with a service in the cathedral, after which the opening meeting was held in the Windsor Hall. The new president, Professor T. G. Roddick, was there formally inducted by Dr. Saundby, president of the Council; and, after the Association had been welcomed by Lord Aberdeen and other high officials, Professor Roddick delivered his address. The same evening Lord Lister was entertained by the Montreal Medico-Chirurgical Society, and a largely attended conversazione was held at Laval University. Thus harmony reigned instead of the wrangles which usually characterise these annual meetings. Exeter Hall had borne the brunt, and Canada was left in peace; science and hospitality being the order of the day.

On Wednesday the sections got fairly to work. There were present 200 members from the United Kingdom and 500 from Canada, while between 200 and 300 physicians and surgeons of the United States were invited to attend as guests.

In the afternoon the McGill University conferred honorary degrees on Lord Lister, Professor Richet, Sir Walter Foster, Sir William Turner, Dr. Barnes, Dr. Michael Foster, Dr. Gaskell, Mr. Christopher Heath, Professor Macalister, Dr. Saundby, and Mr. Wheelhouse.

In the evening a reception in honour of the association was given by Lord Strathcona and Mount Royal, two thousand guests being present.

On Thursday the Mayor and Corporation entertained the visitors at luncheon on the summit of Mount Royal, and in the evening the annual dinner was presided over by Professor Roddick, 500 persons being present.

Garden parties and golf links were visited in the afternoon, and the foundation stone of a new Nurses' Home, in connection with the General Hospital, was laid by Lord Lister.

An end comes to all things, even to meetings of great associations, and on Friday the Montreal meeting of the British Medical Association was brought to a close with an address on "Public Health" by Dr. Herman Biggs, of New York.

The social side of the meeting, which through the hospitality of the Canadians has been especially prominent throughout, ended in the evening by a conversazione at McGill University, which was attended by 3,000 citizens and visitors.

Then came the excursions, which always form so considerable a feature of these meetings. In the meantime let us turn to the consideration of a few of the addresses at these congresses.

### Bacteriological Problems.

In his address on pathology Mr. Watson Cheyne did well to remind us of a fact which some people are but too apt to forget, namely, that we have still much to learn. So all-explanatory have the discoveries of Lord Lister seemed to some, in regard to the relation existing between micro-organisms and suppuration, that people have been ready to believe that, at least so far as suppuration was concerned, their knowledge was complete. But Mr. Watson Cheyne propounds a series of conundrums which may serve to pull down these people's pride. In spite, he says, of all that has been done many problems remain unsolved. In the case of tuberculosis, why is it that in one part of the body we have a slow-growing lupus disease and in another part, perhaps of the same body, a rapidly-developing tuberculosis? The same problem is apparently presented with regard to the bacilli of diphtheria, in that we may have in one case a true diphtheria, in another a membranous rhinitis, while in a third, although the bacilli are present in the throat, the individual may be apparently healthy.

Another very remarkable problem is presented by the results which follow free incisions into tuberculous tissue. An incision is made into the abdominal cavity; masses of tuberculous tissue and tubercle are found scattered over the peritoneum; nothing whatever is done, the wound is stitched up, and yet in many cases the patient, who up to that time has been going steadily down hill, begins to pick up, and the disease may come entirely to a standstill.

Again, what is the meaning of a chronic abscess? How is it that the tubercule bacilli at one time produce a quantity of tuberculous tissue, at another a cheesy mass, and at another a chronic abscess?

In connection with the pyogenic organisms also we have many problems. How is it, for example, that after an abscess is opened antiseptically suppuration at once ceases, seeing that when it is opened it is found to contain living pyogenic organisms? We can easily understand that the subsidence of the fever and general disturbance is due to the diminished absorption of toxic products. But why do n't these living pyogenic organisms keep up the suppuration? and why is it that after two or three days one may fail to obtain any cultivations from the serum which escapes from the wound? These are questions which have no doubt puzzled many people who perhaps have hardly cared to confess that the explanations given were not all-satisfying. It is well that they should be asked in this public way, and that we should recognise how many other factors, beside the mere bacilli, we require to know about before we can feel that we thoroughly understand the subject of suppuration.

Cardiac Disturbance in Relation to Dyspepsia.

In a paper read at Moscow by Dr. Ernest Sansom, on "Neuropathic Dyspepsia and its Correlations with Disturbances of the Rhythm of the Heart," some interesting conclusions were enunciated, the outcome of which are roughly this—that paroxysmal disturbances of rate and rhythm are apt to be connected with dyspeptic disorders, while persistent irregularities and disturbances often are, and so far as concerns tachycardia usually are, quite independent of dyspeptic derangement. Dyspepsia, moreover, does not produce irregularity of beat without some predisposing cause; and, lastly, the dyspepsia which may be found in connection with cardiac disturbance may not be its cause, but merely a further manifestation of the same affection of the nerve element at the origin or in the course of the vagi by which the cardiac mechanism is itself being deranged.

Peritonitis and Appendicitis.

The allied subjects of peritonitis and appendicitis were touched upon at considerable length both at Moscow and at Montreal, a formal discussion on appendicitis having been organised at the latter place.

Speaking at Moscow Professor Roux, of Lausanne, founded his remarks on an experience of 560 of perityphlitis, on which he had had occasion to operate at one stage or another. In upwards of 200 of these cases the operation had been done between the attacks, and had afforded an opportunity of ascertaining the condition in which the vermiform appendix was left after one or more acute attacks of inflammation. The conclusion he had arrived at was that between the various types met with in practice the difference was only one of degree, and that appendicular colic was not a separate disease, the tendency in all cases being towards suppuration and partial or total gangrene. Between the mild cases which had been termed appendicular colic and those serious ones, fortunately rare, in which death resulted from septicæmia and septic peritonitis, there lay the majority of cases, and in these when death resulted it arose from secondary perforation, arising because operation had not been performed in time for the relief of the localised septic peritonitis which usually existed; and he urged that such cases, which were by far the most numerous, should be operated on, even at the home of the patient, by country practitioners, in order to prevent the necessity of removal to a hospital. He said, however, that to insure that the intervention of the surgeon should not be more serious than the disease itself it was necessary not to operate before the abscess became accessible by way of the flank, the rectum, or the vagina, which was usually the case from the fifth to the seventh day. Even in cases in which one could only by percussion perceive a serous or sero-purulent exudation apparently in the peritoneal cavity, cases which simulated general peritonitis so closely that it was easy to make a mistake, if one did but wait a few days the case developed in the same manner as other varieties of perityphlitis, and the abscess might be incised later on through the rectum or the vagina. Prompt operation then should only mean incision when the abscess was accessible without opening the peritoneum, and this should be done by the ordinary practitioner without removal to hospital. Patients suffering from septicæmia and ichorous peritonitis would die just as they would in the hands of a surgeon, but treatment at home would be the means of saving those who were threatened with secondary perforation.

In the interval between the attacks the matter was different. In such cases operation should be done in all cases, for, in spite of all assertions to the contrary, he was convinced that there was really no other means of preventing recurrence.

Dr. Kümmell, of Hamburg, said that since 1889 he had operated for the relief of recurrent appendicitis, in the interval, 103 times, recovery taking place in every case. His conduct during an attack was essentially conservative. Among 400 such cases in which no operation was performed the mortality was only 5 per cent. In recurrent cases he was in the habit of operating after the third attack.

Professor Sonnenburg, of Berlin, however, had observed advanced anatomical lesions in all cases, and thought operation was called for even in the midst of an attack in order to prevent subsequent complications. In 100 cases with a circumscribed purulent focus he had met with no failure.

Dr. Grinda, of Nice, urged the advantages of a lumbo-iliac incision, so as to arrive at the seat of disorder by the safest and most direct route, avoiding interference with the peritoneum, and insuring perfect drainage.

Professor Senn, of Chicago, dealing with the same subject, said that in mild cases of appendicitis from 80 to 90 per cent. recovered under appropriate medical treatment, and that in a fair percentage of the cases the disease did not return. For his own part, he resorted to operation in all cases during a first attack when the symptoms pointed to perforation or gangrene of the appendix. The sooner the operation was undertaken under such circumstances the better were the results. The appendix should be sought for and removed only if pus was found in the iliac fossa, and when it could be done without a material increase in the immediate risks of the operation; otherwise, the treatment by incision and drainage would yield the best results. In relapsing appendicitis operation was indicated, especially where the attacks were repeated at short intervals and with increasing severity.

Drainage of the Peritoneum.

Speaking on the subject of drainage after operations for peritonitis, Professor Senn said that such a proceeding was an admission of the present imperfect state of surgery, and was a confession that the surgeon had not been able to accomplish what he so earnestly desired, namely, complete asepsis of the peritoneal cavity.

There were at present three methods of drainage in general use: (1) tubular drainage; (2) capillary drainage; (3) a combination of tubular and capillary drainage.

Tubular drainage was especially indicated in cases in which the abdominal cavity contained pus. The tubes might be either of glass or of soft rubber, but the latter was justly accused of causing more irritation than the smooth glass tube. Keith's glass tubes served admirably for draining the lowest part of the abdominal cavity. They should be curved at the abdominal end so as to reach the floor of the pelvic cavity without making harmful pressure against the bladder. Frequent aspiration of the contents of the drain was necessary for the purpose of removing the fluid inflammatory products as soon as they were formed. Prolonged tubular drainage was apt to cause intestinal fistula by the pressure produced, and to prevent this he was in the habit of surrounding the glass or rubber tube with a few layers of iodoform gauze securely fastened to the tube.

Capillary drainage was frequently employed as a substitute for that by tubes, and also must often be relied upon as an important hæmostatic resource in arresting parenchymatous oozing. The gauze drainage devised by Mikulicz had proved of great value in the treatment of peritonitis. The typical Mikulicz tampon was made by taking a piece of iodoform gauze the size of a large handkerchief, to the centre of which a strong piece of silk thread was stitched. When used it was arranged as a pouch, and carried by means of curved forceps to the bottom of the pelvis and filled with strips of iodoform gauze, the free end of the silk thread issuing from the mouth of the pouch. When it was desired to remove the drain the gauze strips were drawn out, and the pouch was removed by making traction upon the string. In dealing with large cavities, however, requiring an enormous amount of gauze, he had learned to fear iodoform gauze because the cases were by no means isolated, in which a drain composed exclusively of it had become the immediate cause of death from iodoform intoxication, which was specially likely to happen when the functions of the kidney were being imperfectly performed. He would, therefore, limit the iodoform gauze to an outer layer or two, and pack the pouch with ordinary sterilised gauze.

Drainage by sterilised wicking had been popular in Germany for a number of years, and in many cases had answered well. R. T. Morris employed wicks in a peculiar way. The simplest consisted of a little roll of perchloride gauze, round which was wrapped a couple of layers of Lister's protective silk. The gauze protruded a little from each end of the cylinder, and a few small fenestræ in the protective silk allowed the serum to reach the gauze elsewhere. Where a large gauze packing for the pelvis or abdomen was needed, an apron of the silk could expand over the gauze so as to prevent the occurrence of intestinal adhesions. This method possessed great advantages over ordinary tubular and capillary drainage, and recommended itself more especially in the surgical treatment of diffuse septic peritonitis. The prolonged contact of gauze with a serous surface was very prone to give rise to permanent adhesions.

In employing gauze in draining the general peritoneal cavity it was necessary to use long strips, which should be inserted in different directions and brought out at the same place, and fastened together with a safety pin. It had been shown by Van Hook that gauze drained more freely if the external ends were left long and placed on the side of the pelvis below the level of the wound. Drainage must always be dispensed with as soon as possible, in order to prevent adhesions and to enable the surgeons to close the incision by secondary suturing, an important precaution against the formation of a ventral hernia. The strips should be shortened, and one after the other removed as the indications for drainage disappeared.

A useful plan in some cases was to combine the tubular and the capillary drain by packing loosely a glass drain of proper length and size with strips of gauze or aseptic wicking. This was of especial service when the inflammatory product was of serum instead of pus.

Tuberculosis.

As was to be expected, a good deal of stress has been laid upon tuberculosis in the various discussions. Professor Lannelongue, of Paris, speaking of its surgical treatment, laid especial emphasis on the necessity of early and complete removal of the disease whenever this was practicable. He spoke of the local centres of infection as threatening the whole organism, and insisted on their removal. Objections of various sorts had been raised to this line of argument, but he thought they would not bear criticism. Especially it had been objected that the tubercle bacilli were accompanied by other micro-organisms, the action of which would continue notwithstanding the extirpation of the tuberculous forms of disease. He thought, however, that the presence of these other organisms was a secondary result of the tuberculosis, and wherever it was possible to effect removal the surgeon should act thoroughly, and should act at once.

Where it was not possible to remove the disease he advocated the method of building up a wall of firm and hardened tissue around the diseased patch, and so shutting off the pathological focus from any communication with the healthy structures around. This sclerogenic treatment he carried out by injecting irritating substances deeply into the parts surrounding the diseased centre, by which means he was able to cause the production of an envelope of fibrous tissue enclosing the tuberculous focus. In addition to producing this fibrous tissue, the injection caused a great afflux of leucocytes, whose phagocytic action contributed to the destruction of the bacilli. The experiments of Nocard had demonstrated that the tubercle bacilli, when enclosed in this sclerotic zone, were all killed in from six to eight weeks.

A paper by Professor Leyden, of Berlin, speaking rather of medical tuberculosis, and evidently referring chiefly to pulmonary phthisis, attacked the question from a very different standpoint. Either, he said, the bacillus might be attacked directly, or the constitutional powers of the individual might be so reinforced as to render him able to withstand the evil influence of the bacilli. The attempts which had been made by Koch to produce a curative tuberculin had not, he thought, been crowned with success, and, until better results could be obtained in that direction, the best plan would be to treat such patients in specially-constructed sanatoria, where alone all the various requirements for a successful scheme of treatment could be carried out.

Speaking in another section, Dr. Petit, of Paris, also advocated a prophylactic treatment of pulmonary tuberculosis by the establishment of sanatoria for those suffering from that disease.

It should be noted, however, that the congress received a communication from Dr. Petruschki, giving notes of two cases in which the tuberculin of Koch had been successfully used.

Perhaps there is too great a tendency for people to divide themselves into camps on this question of tuberculosis, for it is clear that even the extremest measures for the local removal of the disease by the surgeon in no way take away the necessity for that hygienic treatment which has been found by physicians to be of itself successful in some cases in which operative proceedings have been out of the question.

The whole question of the prevention of tuberculosis in crowded communities was dealt with at Montreal in an interesting address by Dr. Hermann Biggs, of which we propose to give some account next week.

